# hnRNP I regulates neonatal immune adaptation and prevents colitis and colorectal cancer

**DOI:** 10.1371/journal.pgen.1006672

**Published:** 2017-03-15

**Authors:** Zhigang Jin, Feng Liang, Jing Yang, Wenyan Mei

**Affiliations:** 1 Department of comparative Biosciences, College of veterinary medicine, University of Illinois at Urbana-Champaign, Urbana, Illinois, United States of America; 2 Department of statistics, University of Illinois at Urbana-Champaign, Champaign, Illinois, United States of America; St Jude Children's Research Hospital, UNITED STATES

## Abstract

The intestinal epithelium plays a critical role in host-microbe homeostasis by sensing gut microbes and subsequently initiating proper immune responses. During the neonatal stage, the intestinal epithelium is under immune repression, allowing the transition for newborns from a relatively sterile intra-uterine environment to one that is rich in foreign antigens. The mechanism underlying such immune repression remains largely unclear, but involves downregulation of IRAK1 (interleukin-1 receptor-associated kinase), an essential component of toll-like receptor-mediated NF-κB signaling. We report here that heterogeneous nuclear ribonucleoprotein I (hnRNPI), an RNA binding protein, is essential for regulating neonatal immune adaptation. We generated a mouse model in which hnRNPI is ablated specifically in the intestinal epithelial cells, and characterized intestinal defects in the knockout mice. We found that loss of hnRNPI function in mouse intestinal epithelial cells results in early onset of spontaneous colitis followed by development of invasive colorectal cancer. Strikingly, the epithelium-specific hnRNPI knockout neonates contain aberrantly high IRAK1 protein levels in the colons and fail to develop immune tolerance to environmental microbes. Our results demonstrate that hnRNPI plays a critical role in establishing neonatal immune adaptation and preventing colitis and colorectal cancer.

## Author summary

Precisely controlled host-microbe interactions in the gastrointestinal tract are crucial for human overall health and well-being. Dysregulated host responses to gut microbiota are the major cause of autoimmune diseases, inflammatory disorders and cancers. The intestinal epithelium lines the gastrointestinal tract and plays a critical role in sensing gut microbes and subsequently developing a balance of immune tolerance and active immune responses. During the neonatal stage, the immune system in the gastrointestinal tract must be temporally suppressed to accommodate the large number of newly arrived microbes. This process is known as neonatal immune adaptation, and is critical for the establishment of proper host- microbe interactions. We studied the function of hnRNPI in the intestinal epithelium by genetically ablating it in the intestinal epithelial cells of mouse. We found that loss of hnRNPI in intestinal epithelial cells disrupts neonatal immune adaptation, resulting in spontaneous colitis and early onset of invasive colorectal cancer. We show that hnRNPI is required for the neonatal immune suppression through decreasing the protein level of IRAK1, an essential component of toll-like receptor-mediated NF-κB signaling. Our studies demonstrate a critical role of hnRNPI in establishing neonatal immune adaptation and preventing colitis and colorectal cancer.

## Introduction

Increasing evidence indicates that proper host-microbe interaction in the gastrointestinal tract is critical for the balance of immune tolerance and active immune responses [1,2]. Dysregulated host response to gut microbiota is the major cause of autoimmune diseases, inflammatory disorders and cancer [1,3–6]. The intestinal epithelium, which lines the gastrointestinal tract, plays a fundamental role in controlling the host-microbe interaction [7]. Structurally, the intestinal epithelium acts as a physical barrier to separate luminal contents from immune cells situated in the lamina propria. A mucus layer formed by goblet cells covers the intestinal epithelium and protects it from direct attack of foreign antigens [8]. Moreover, junction complexes located between the intestinal epithelial cells (IECs) control the paracellular permeability of the intestinal epithelium, which is critical to prevent the invasion of pathogens and other luminal contents across the epithelial layer [9,10]. In addition to acting as a physical barrier, IECs play an active role in immune defense by expressing a variety of molecules that recognize and subsequently kill pathogens, and initiating the innate and adaptive immune responses. One of the most important pathways that act in IECs is Toll-like receptor (TLR) -mediated NF-κB signaling. A number of TLRs are expressed in the IECs [11]. Upon binding with their ligands, which are the conserved molecular motifs on microorganisms, TLRs activate a series of downstream signaling cascades and subsequently activate NF-κB signaling [11–13]. A key event in transmitting signals from TLRs to NF-κB signaling is IRAK1-induced degradation of IκB, the cytosolic inhibitors of NF-κB signaling. This consequently releases NF-κB subunits from a cytoplasmic inhibitory complex, which allows them to translocate into the nucleus to induce transcription of pro-inflammatory genes. Prolonged or excessive TLR-mediated NF-κB signaling activation is a major cause of inflammatory disorders and inflammatory bowel disease-associated colorectal cancer [14–17]. Thus, understanding mechanisms by which TLR -mediated NF-κB signaling is precisely controlled in the IECs is critical for elucidating the etiology of gastrointestinal inflammatory disorders and its associated cancers.

Recent studies indicate that TLR -mediated NF-κB signaling is suppressed in the intestinal epithelium during the neonatal stage [18,19]. Upon birth, newborns undergo a transition from a sterile intra-uterine environment to one that is rich in environmental microbes. To accommodate the colonization of the commensal intestinal microorganisms, the intestinal epithelium of the newborn undergoes a series of dynamic changes in gene expression to suppress the TLR -mediated NF-κB signaling activity. One of the most important events is downregulation of the IRAK1 protein level in the IECs shortly after birth [18]. This downregulation is at least partly through TLR4 signaling-mediated continuous proteolytic degradation of IRAK1 during the neonatal stage [19]. A low level of IRAK1 protein in IECs is essential for inhibiting excessive immune response to newly arrived gut microbes and facilitating microbe colonization in the neonate [18,19]. Recent studies show that miR-146a is essential to maintain the low level of IRAK1 protein in the neonatal IECs [19]. However, the detailed molecular mechanism by which IRAK1 is downregulated by miR-146a remains elusive. In addition, it is unclear whether other inhibitory mechanisms are involved during neonatal immune adaption.

We previously reported that hnRNPI, an RNA binding protein, is an important regulator of intestinal epithelium renewal and calcium-mediated egg activation in zebrafish [20,21]. hnRNPI, also known as polypyrimidine tract-binding protein (PTB), plays important roles in alternative splicing and other post-transcriptional regulatory events [22,23]. A number of hnRNPI targets are abnormally spliced in intestinal inflammatory and neoplastic diseases [24–31], suggesting that hnRNPI-dependent post-transcriptional control may play important roles in pathogenesis of these diseases. To determine hnRNPI functions in mammalian intestinal homeostasis and more importantly, to understand how malfunction of this protein contributes to inflammation and colorectal cancer, we have generated IEC-specific hnRNPI knockout mice. We show here that ablation of hnRNPI in the IECs induces spontaneous colitis in mice followed by development of invasive colorectal cancer at a young age. We further show that inflammation occurs shortly after birth in the knockout neonate, which is accompanied by hyperactive NF-κB signaling in the colonic epithelial cells. We provide evidence that downregulation of IRAK1 protein expression is disrupted in the knockout neonatal colon, whereas expression levels of TLRs remain unaffected. Thus, our results reveal a novel role of hnRNPI in establishing neonatal immune adaptation, which is at least partly through the control of the IRAK1 protein level.

## Results

### Generation of a mouse model in which hnRNPI is ablated specifically in IECs

**Fig 1:**
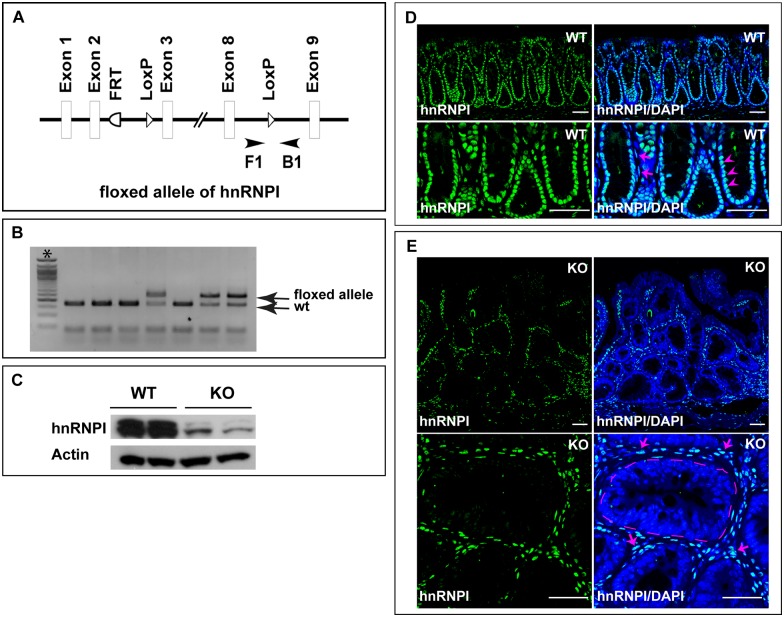
Generation of the IEC-specific hnRNPI knockout mice. (A) A schematic representation shows the knockout strategy. A primer pair (F1 and B1) flanking one loxP site (arrowheads) was used for genotyping the IEC-specific hnRNPI knockout mice. (B) An example of genotyping PCR result shows the sizes of PCR products amplified from the hnRNPI floxed allele (236 bp) and wild-type allele (166 bp). *, 50bp DNA ladder. (C) Western blotting result shows efficient ablation of hnRNPI in the colonic epithelial cells of the knockout mice. hnRNPI protein levels from two knockout mice and two control sibling littermates are shown. (D) and (E) Immunofluorescence staining using an anti-hnRNPI antibody shows hnRNPI protein localization in the wild-type and hnRNPI knockout colon. Magnified images are shown in the lower panels. Nuclear accumulation of hnRNPI protein was detected in both colonic epithelial cells (arrowheads) and cells in the lamina propria (arrows) in wild-type mice (D). hnRNPI expression is diminished in the nuclei of colonic epithelial cells in the knockout mice, but remains unaffected in the lamina propria cells (E, arrows). Note the increased number of immune cells in the lamina propria of the knockout mice. The dotted line indicates the border of a centrally located crypt. Nuclei were counterstained with DAPI. WT, wild-type; KO, knockout. Scale bars, 50 μm.

Our previous studies in the zebrafish hnRNPI mutant indicate that hnRNPI plays a key role in balancing IEC proliferation and differentiation [20]. To determine the functions of hnRNPI in mammalian intestinal homeostasis, we examined its expression in the mouse intestine. We found that hnRNPI protein is highly accumulated in the nuclei of IECs as well as cells situated in the lamina propria (Fig 1D and 1E, S2–S5 Figs). To determine the role of hnRNPI in the mammalian IECs, we generated a floxed mouse allele of hnRNPI, in which two loxP sites flank the DNA region of exon 3 to exon 8 of the hnRNPI locus (Fig 1A and 1B). This allows deletion of the three most abundant isoforms of hnRNPI upon the Cre recombination [32]. By breeding the hnRNPI floxed allele with a Cre line controlled by the *villin* promoter [33], we generated the *hnRNPI*^*flox/flox*^; *Villin*^*Cre/+*^ (hereafter IEC-specific hnRNPI knockout) mice. The villin promoter directs expression of the Cre recombinase in the IECs as early as at embryonic day 12.5 [33], which allows epithelium-specific deletion of hnRNPI at late embryogenesis in the knockout mice. As expected, the expression of hnRNPI protein was dramatically downregulated in the IECs of the hnRNPI knockout mice, but not that of the control mice (Fig 1C–1E).

### The IEC-specific hnRNPI knockout mice develop spontaneous colitis

**Fig 2:**
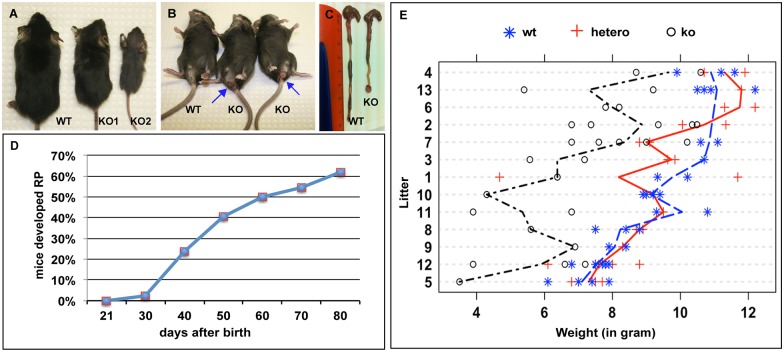
The IEC-specific hnRNPI knockout mice have low body weight and develop rectal prolapse. (A) Size difference between the knockout mice (KO) and their control littermate (WT) at the time of weaning. The knockout mouse labeled as KO2 weighted 50% less than the control mice and died within three days after weaning. (B) The knockout mice develop rectal prolapse (arrows). (C) Gross morphology of the colons from a knockout mouse and its control littermate. The mutant colon is short with prolapsed rectum. (D) Timeline of rectal prolapse development in the knockout mice within 80 days after their birth (n = 41). (E) Statistical analysis using R shows low body weights of the adult knockout mice when compared to those of their control littermates. Total 13 litters (100 mice) aged between day 20 to day 28 from the cross of *hnRNPI*^*flox/+*^; *Villin*^*Cre/+*^ mice with the *hnRNPI*^*flox/flox*^ mice were analyzed. “*”, “+”, and “o” in the graph indicate the genotype and weight of the individual mouse analyzed. The weight differences between ko and wt, or ko and hetero, are all statistical significant with p-values less than 0.001. The difference between the two control groups (hetero vs wt) is not significant. wt, wild-type, which includes *hnRNPI*^*flox/+*^ mice and *hnRNPI*^*flox/flox*^ mice; hetero, heterozygote (*hnRNPI*^*flox/+*^; *Villin*^*Cre/+*^); ko, knockout (*hnRNPI*^*flox/flox*^; *Villin*^*Cre/+*^).

The IEC-specific hnRNPI knockout mice were born at the Mendelian ratio, but appeared smaller than their littermates (Fig 2A). Their body weight at weaning is significantly less than that of their wild-type littermates (Fig 2E). Severely affected mutants, which weighed 50% less than wild-type littermates, died within three days after weaning, likely due to their malfunction in digesting solid food (mouse labeled as KO2 in Fig 2A is representative). Among the remaining knockout mice, over 60% of them developed rectal prolapse within 80 days after birth (Fig 2B–2D, n = 41).

**Fig 3:**
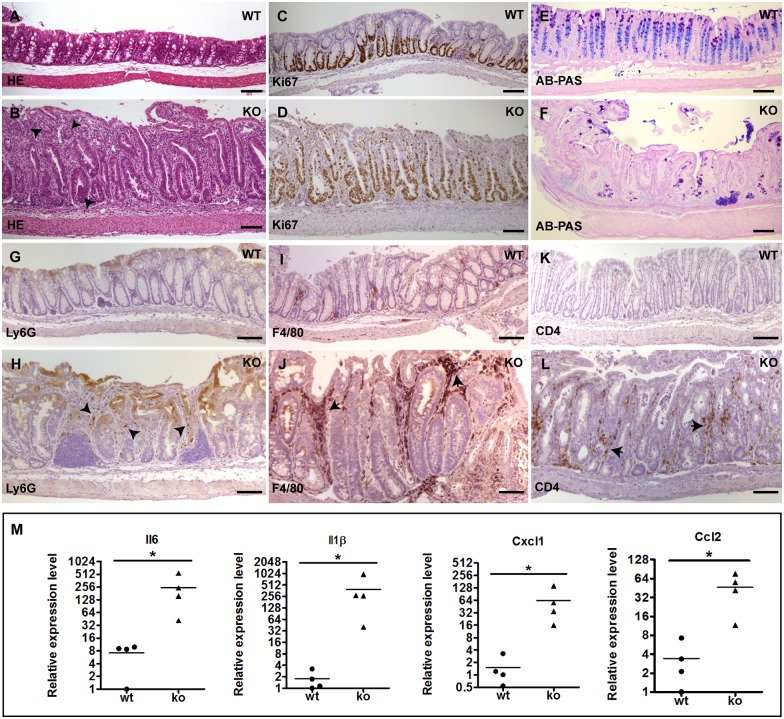
The IEC-specific hnRNPI knockout mice develop spontaneous colitis. (A) and (B) Hematoxylin and Eosin (HE)–stained sections show severe inflammation in the colonic epithelium of the knockout mice (B). Arrowheads in B show crypt abscesses. (C) and (D) Immunohistochemical staining with an anti-Ki67 antibody shows hyperproliferation of colonic epithelial cells in the knockout mice (D). (E) and (F) Alcian Blue-PAS staining shows loss of goblet cells in the colonic epithelium of the knockout mice (F). (G) to (L) Immunohistochemical staining shows increased number of inflammatory cells in the lamina propria of the knockout colon with indicated antibodies. (G) and (H) show neutrophils. Arrowheads in H point neutrophils in the knockout mice. (I) and (J) show macrophages. Arrows in J point macrophages in the knockout mice. (K) and (L) show CD4 positive T-cells. Arrows in L point CD4 positive T-cells in the knockout mice. (M) Real-time PCR shows increased expression of the pro-inflammatory cytokines and chemokines in the colonic epithelial cells of the knockout mice. Each symbol in all graphs indicates gene expression level relative to *Gapdh* of individual mice. Bars show mean value. In both the wild-type and knockout groups, n = 4 mice. * p< 0.05. Scale bars, 50 μm.

We performed histological analysis on the 3-week to 12-week old knockout mice and found that 100% of them developed moderate to severe degree of inflammation in the colonic epithelium and their colons often appeared shortened (Fig 2C, n = 26, 13 of them < 4 weeks). There is no gender-based difference in the development of colon inflammation in these mice. Histological features of the inflamed colonic epithelium in the knockout mice include crypt elongation and abscesses, loss of goblet cells, inflammatory cell infiltrate, and impaired surface integrity (Fig 3A, 3B, 3E and 3F). This is accompanied by hyperproliferation of IECs (Fig 3C and 3D). Large numbers of infiltrated inflammatory cells including Ly6G positive neutrophils, F4/80 positive macrophages, and CD4 positive T cells, were detected in the lamina propria (Fig 3G–3L). These phenotypes highly resemble the pathological features of human ulcerative colitis. Mice with a lower body weight displayed more severe colitis. While the colitis phenotype was observed in all parts of colon in the knockout mice, epithelium in the distal colon is more severely affected. In contrast, the epithelium of the small intestine did not display histologically detectable inflammation in these mice.

In line with the morphological appearance of colitis in the knockout mice, the expression of proinflammatory cytokines and chemokines, including IL6, IL1β, Cxcl1, and Ccl2, are dramatically increased in the colonic epithelial cells of the knockout mice (Fig 3M). Thus, IEC-specific depletion of hnRNPI results in early onset of spontaneous colitis.

### The IEC-specific hnRNPI knockout mice develop neoplasia and invasive colorectal cancer at a young age

**Fig 4:**
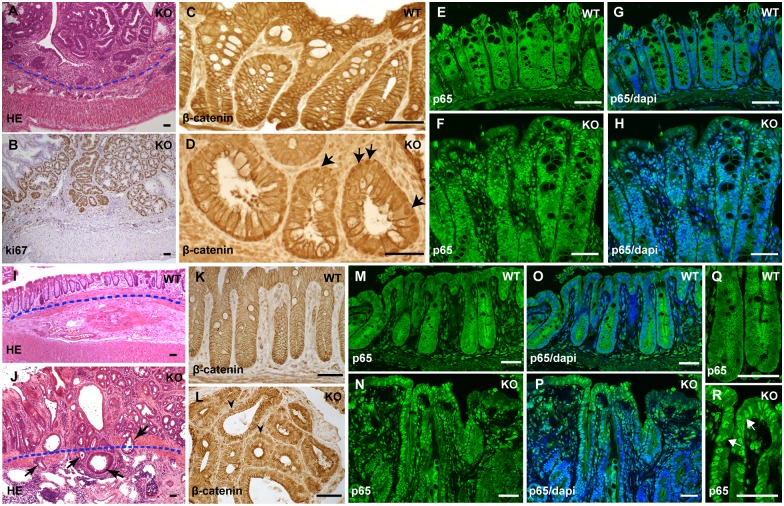
The IEC-specific hnRNPI knockout mice develop colorectal neoplasia with highly active Wnt and NF-κB signaling. (A) HE–stained section shows non-invasive neoplasia in the colonic epithelium of the knockout mouse. The dotted line indicates the position of muscularis mucosae. (B) Immunohistochemical staining with an anti-Ki67 antibody shows hyperproliferation in colonic neoplasia. (C) and (D) Immunohistochemical staining with an anti-β-Catenin antibody shows nuclear translocation of β-Catenin in the colonic epithelium of the knockout mice (D, arrows). In the wild-type colonic epithelium, β-Catenin is membrane localized (C). (E) to (H) Immunofluorescence staining with an anti-p65 antibody shows p65 nuclear translocation in the colonic epithelium of the knockout mice (F and H). p65 is mainly localized in the cytoplasm in the control mice (E and G). Nuclei were counterstained with DAPI (G and H). (I) and (J) HE–stained section shows invasive adenocarcinomas in the prolapsed rectum of the knockout mouse. The dotted line indicates the position of muscularis mucosae. Note a few glands have invaded through the muscularis mucosae into the submucosa (arrows in J). (K) and (L) Immunohistochemical staining shows nuclear accumulation of β-Catenin in the rectal epithelial cells in the knockout mice (arrowheads in L), whereas in the control rectal epithelium, β-Catenin is cell membrane localized (K). (M) to (R) Immunofluorescence staining shows nuclear translocation of p65 in the rectal epithelium of the knockout mice (N, P and R). The control mice showed cytoplasmic localization of p65 (M, O and Q). Nuclei were counterstained with DAPI (O and P). (Q) and (R) High magnification shows p65 nuclear localization in the rectal epithelial cells. WT, wild-type; KO, knockout. Scale bars, 50 μm.

Intriguingly, we observed multiple adenomatous lesions in the colonic epithelium of the knockout mice. We analyzed total 30 mice aged between P22 to P230, and found 60% of them developed colon adenomas at variable degrees of dysplasia. Among them, the youngest mice that had adenomas were at the age of P23. These lesions display hyperproliferation of colonic epithelial cells (Fig 4A and 4B). Nuclear accumulation of β-catenin and p65, the respective hallmarks of active Wnt signaling and NF-κB signaling, is prominent in the lesions (Fig 4C–4H). This indicates that colon adenomatous lesions in these mice are in the precancerous condition. All adenomatous lesions in the colonic epithelium, however, are restricted to the mucosa (Fig 4A). In striking contrast, we found that lesions developed in the epithelium of the prolapsed rectum were highly invasive, and had spread through the muscularis mucosae into the submucosa (compare Fig 4J to 4I). We examined total 17 knockout mice aged between P42 to P230 that developed rectal prolapse, and found 15 of them (88%) developed invasive adenocarcinomas in the rectal epithelium. Among those with rectal adenocarcinomas, the youngest knockout mouse was at the age of P50. Similar to the lesions in the colon, these rectal carcinomatous lesions contain highly active Wnt signaling and NF-κB signaling (Fig 4K–4R).

### The IEC-specific hnRNPI knockout mice develop colonic inflammation postnatally

**Fig 5:**
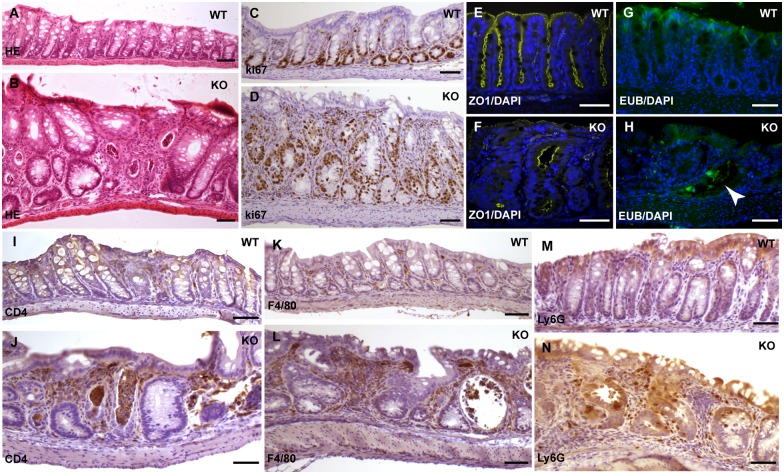
The IEC-specific hnRNPI knockout mice develop severe inflammation at P14. (A) and (B) HE–stained sections show the severely inflamed colonic epithelium in the knockout mice at P14 (B). (C) and (D) Immunohistochemical staining with an anti-Ki67 antibody shows hyperproliferation of the colonic epithelium in the knockout mice. (E) and (F) Immunofluorescence staining with an anti-ZO1 antibody shows impaired tight junctions in the colonic epithelium of the knockout mice. (G) and (H) FISH with a universal eubacterial probe shows bacteria infiltration in the colonic epithelium of the knockout mice (arrowhead in H). Nuclei were counterstained with DAPI in E to H. (I) to (N) Immunohistochemical staining with indicated antibodies shows increased number of inflammatory cells in the lamina propria of the knockout mice. (I) and (J) show CD4 positive T-cells, (K) and (L) show macrophages, (M) and (N) show neutrophils. WT, wild-type; KO, knockout. Scale bars, 50 μm.

To determine when colitis development in the IEC-specific hnRNPI knockout mice is initiated, we performed histological analysis on the colonic epithelium from the knockout mice and their sibling littermates at P7 and P14. Severe inflammation was observed in the colonic epithelium of the knockout mice at both P7 and P14 (the histology of wild-type and knockout colon at P14 is shown in Fig 5A and 5B respectively). At P14, the colonic epithelium of the knockout mice displayed impaired intestinal epithelium junctional complexes as shown by the zonula occludens (ZO)-1 staining (compare Fig 5F to 5E). Consistently, we detected bacteria infiltration in the mutant intestinal epithelium (compare Fig 5H to 5G), indicating destruction of the epithelial barrier. Furthermore, a large number of infiltrated innate and adaptive immune cells were seen in the lamina propria (Fig 5I–5N). These defects are accompanied by hyperproliferation of IECs (compare Fig 5D to 5C).

**Fig 6:**
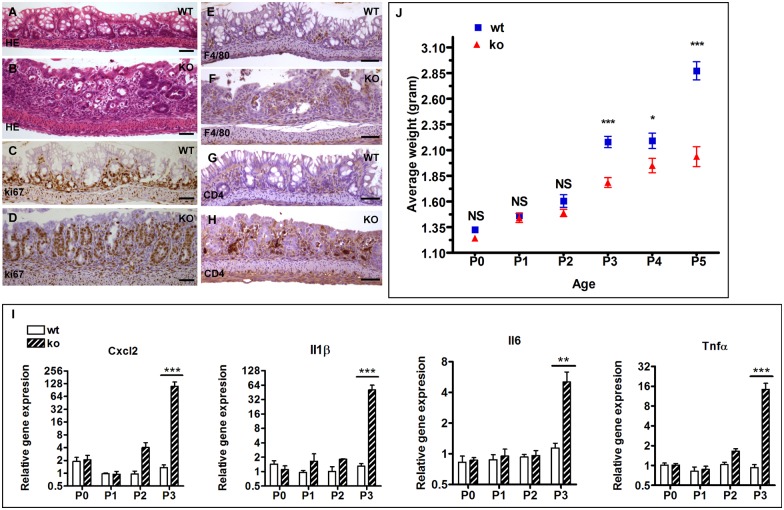
The IEC-specific hnRNPI knockout mice display colonic inflammation and weight gain decline at P3. (A) and (B) HE–stained sections show the inflamed colonic epithelium in the knockout neonates (B). (C) and (D) Immunohistochemical staining with an anti-Ki67 antibody shows hyperproliferation of the colonic epithelial cells in the knockout neonates. (E) to (H) Immunohistochemical staining with indicated antibodies shows increased number of inflammatory cells in the lamina propria of the knockout neonates. (E) and (F) show macrophages, and (G) and (H) show CD4 positive T-cells. (I) Representative real-time PCR results show significantly increased expression of pro-inflammatory cytokines and chemokines in the knockout colon at P3. Data are presented as mean values relative to *Gapdh* (± s.e.m). N = 3–5 mice per group. Mice of the knockout group and the wild-type group are sibling littermates. Mice from at least 3 litters were tested at each age. (J) Starting at P3, the knockout neonates display decline in the weight gain. Each age contains at least 6 litters, and total 47 litters of 367 mice were analyzed. Data are presented as mean values ± s.e.m. * p < 0.05; ** p < 0.01; *** p < 0.001; NS, not significant. WT, wild-type; KO, knockout. Scale bars, 50 μm.

We further examined the colonic epithelium during the first week of life. While the colonic epithelium from the knockout neonates appear histologically normal at P0, P1 and P2 (the colon histology of P2 neonates is shown in S1 Fig), inflamed colonic epithelium was readily observed in the knockout neonates at P3 (compare Fig 6B to 6A). Significant increase in the numbers of proliferating epithelial cells and inflammatory cells was detected in the knockout neonates at P3 (Fig 6C–6H). Consistent with the histological observation, the expression levels of proinflammatory cytokines and chemokines including IL6, IL1β, TNF**α** and Cxcl2 are dramatically upregulated in the colon of the knockout mice at P3, while those at P0 and P1 remain unchanged (Fig 6I). At P2, a slight increase in the expression of Cxcl2, IL1β, and TNF**α** was detected in the knockout colon (Fig 6I). It appears that the immune response was initiated at the molecular level at P2 in these mutants.

Interestingly, we observed a tight correlation of colonic inflammation onset and decline in weight gain in the knockout neonates. As shown in Fig 6J, a sharp decline in the weight gain occurred in the knockout neonates at P3, a time point when colonic inflammation was first observed histologically.

### Ablation of hnRNPI in the IECs induces hyperactive NF-κB signaling and upregulation of the IRAK1 protein level in the neonatal colon

**Fig 7:**
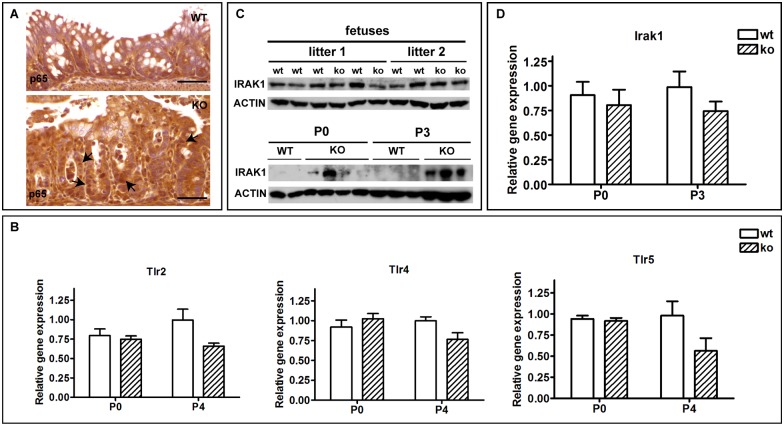
The IEC-specific hnRNPI knockout neonates display hyperactive NF-κB signaling and upregulation of IRAK1 protein expression in the colon. (A) Immunohistochemical staining with an anti-p65 antibody shows p65 nuclear translocation in the colonic epithelium of the knockout mice at P3 (the bottom panel, arrows). (B) Real-time PCR results show that the expression of TLRs is not increased in the colon of the knockout mice at both P0 and P4. (C) Western blot shows IRAK1 protein expression is increased in the knockout colon at P0, and the increase of IRAK1 protein level is more prominent at P3. The IRAK1 protein level remains unchanged in the knockout fetal colon. Actin is served as the loading control. At least 3 litters of each age were tested and data shown are representative. (D) Real-time PCR result shows that the mRNA level of IRAK1 in colons of the knockout neonates is not upregulated at both P0 and P3. Data in B and D are presented as mean values relative to *Gapdh* (± s.e.m). N = 3–5 mice per group. The knockout group and the wild-type group are sibling littermates. At least 3 litters of each age were tested and data shown are representative. WT, wild-type; KO, knockout. Scale bars, 50 μm.

In IECs, TLRs mediated NF-κB signaling plays an essential role in sensing luminal bacteria and initiating subsequent immune reactions [11]. During the neonatal stage, activity of NF-κB signaling is suppressed transiently, allowing microbe colonization and development of immune tolerance. This occurs at least in part through downregulating the expression of IRAK1 protein in the IECs [18,19]. Given the aforementioned hyperactive inflammatory responses in the knockout neonatal colon, we examined the activity of NF-κB signaling in the neonatal colonic epithelium by assessing the cellular localization of the NF-κB subunit p65. Indeed, whereas p65 is localized to the cytoplasm of colonic epithelial cells in the wild-type control mice, nuclear translocation of p65 is prominent in the knockout colon (compare the top and bottom panels in Fig 7A). This observation prompted us to determine whether hyperactive NF-κB signaling in the colonic epithelial cells of the knockout neonates is due to altered expression levels of TLRs and/or IRAK1. We first examined mRNA expression levels of TLR2, 4, and 5, three TLRs that are known to be expressed in the mouse colon [11]. No increase in mRNA expression levels of TLR2, 4, and 5 was detected in the knockout mice at both P0 and P4 (Fig 7B). In striking contrast, increased protein expression of IRAK1 was detected in the colon of the knockout neonates at P0 (Fig 7C). This increase was more dramatic by P3 (Fig 7C). Notably, IRAK1 protein expression in the knockout fetuses remains unchanged (Fig 7C), indicating that hnRNPI is required to downregulate IRAK1 protein level postnatally. Interestingly, we found that the mRNA expression level of IRAK1 was not upregulated in the knockout colon at both P0 and P3 (Fig 7D). This suggests that hnRNPI regulates IRAK1 at the post-transcriptional level. Taken together, the above observations demonstrate that hnRNPI plays an essential role in downregulating expression of IRAK1 protein in the neonatal colon and is essential for neonatal immune adaptation.

## Discussion

Precisely controlled host-microbe interactions are crucial for human overall health and well-being. Neonatal immune adaptation is the first and fundamentally important step in establishing host- microbe homeostasis. During the neonatal stage, the innate immune activity in the digestive tract must be temporally suppressed to accommodate the large number of newly arrived microbes. Recent studies show that this temporal suppression is at least in part through downregulating the expression of IRAK1 protein in the IECs upon birth, a process that requires the presence of miR-146a in the neonatal IECs [19]. The mechanism by which miR-146a downregulates IRAK1 is unknown. It is also unclear if other mechanisms are involved in this process during neonatal immune adaptation.

Here, we report that deletion of hnRNPI in the IECs impairs downregulation of IRAK1 in the neonatal colon. We show that the expression level of IRAK1 protein, but not its mRNA, is upregulated in the neonatal colon upon deletion of hnRNPI in the IECs, suggesting that hnRNPI-mediated IRAK1 downreulation occurs at the post-transcriptional level. Interestingly, IEC-specific deletion of hnRNPI does not affect the protein level of IRAK1 in the fetal colon. These findings are consistent with the recent observation that IRAK1 is downregulated in the neonatal intestine through post-transcriptional regulation, and this process requires microbial stimulation at birth and postnatally [18,19]. In line with the finding that deletion of hnRNPI increases IRAK1 expression in the neonatal colon, we found that NF-κB signaling is highly active in the neonatal colon of the mutant mice. This is accompanied by the induction of colonic inflammation in the knockout neonates, which becomes detectable histologically and molecularly within the first three days after birth. The timing of colon inflammatory response is coincident with the transition of neonates from a sterile intra-uterine environment to one that is rich in foreign antigens, suggesting that mutant neonates fail to develop immune tolerance. We also observed a significant decline in weight gain in the hnRNPI mutant neonates at P3, which is concomitant to the induction of colon inflammation. It is highly likely that the slow weight gain in the mutant neonates is caused by malnutrition in these mice due to the impaired host-microbe interactions. Collectively, these findings uncover an important function of hnRNPI in suppressing the expression of IRAK1 protein in the neonatal colon and establishing host-microbe homeostasis upon birth in the intestine.

Mechanistically, how does hnRNPI regulate the expression of IRAK1? Several splicing variants of IRAK1 with variable stability and activity in mediating TLR-induced NF-κB signaling have been identified in mice and humans [34–37]. It is tempting to speculate that hnRNPI may down-regulate IRAK1 through regulating alternative splicing of IRAK1 or its upstream regulators. Alternatively, hnRNPI, which has the ability to regulate translational efficiency through binding 3’ UTR of its targets [23,38], may directly repress IRAK1 translation, or indirectly alter the translation of its regulators. Interestingly, the 3’ UTR of IRAK1 contains multiple sites resembling the consensus hnRNPI binding sequences. It will be of great interest to determine if hnRNPI physically interacts with IRAK1 3’UTR and regulates IRAK1 translation. Down-regulation of IRAK1 in the neonatal intestine requires continuous proteasome or lysosome-dependent proteolytic degradation [19]. Thus, it is also possible that hnRNPI may regulate the expression of proteins that alter IRAK1 protein turnover. Of note, hnRNPI is capable of modulating the activity of microRNAs in disease pathogenesis and many important biological processes [30,39–42]. It has been reported that miR-146a controls both translation and degradation of epithelial IRAK1 in the neonatal intestine [19]. It would be interesting to determine if hnRNPI regulates the biosynthesis or activity of miR-146a and/or other microRNAs in establishing neonatal immune tolerance. Further studies are required to distinguish these possibilities.

Our results reveal that hnRNPI-deficient mice develop invasive colorectal cancer at a very young age (as early as at P50). This observation is consistent with the findings that hnRNPI is aberrantly expressed in colorectal cancer cells [24,31], and a number of hnRNPI targeting genes are abnormally spliced in colorectal cancer [24–30]. While it is possible that the colorectal cancer development in the hnRNPI-deficient mice may be a consequence of impaired neonatal host-microbe homeostasis, it is more likely that hnRNPI plays additional roles in preventing colitis and colorectal cancer development in adulthood. In agreement with this view, we found that Wnt signaling, a major driver of colorectal cancer, is hyper-active in the hnRNPI-deficient colonic epithelial cells. It has been reported that Wnt ligands are expressed in the IECs and intestinal stromal cells [43,44]. Stromal cells-derived Wnts, but not epithelial cells-produced Wnts, are indispensable for intestinal homeostasis [45]. We thus assessed the expression of six Wnts that are expressed in the colonic stroma [43,44]. These include Wnt2b/Wnt4/Wnt5a, which are highly expressed in the colon mesenchyme, and Wnt5b/Wnt10b/Wnt16 that are expressed at low levels [43,44]. We observed a trend of increase in the expression of Wnt2b in the hnRNPI-deficient mice (S6 Fig). However, this increase is not statistically significant. We did not detect any statistically significant changes in the expression of Wnt4, Wnt5a, and Wnt5b (S6 Fig). In the case of Wnt10b and Wnt16, the expression was decreased (S6 Fig). Currently we do not understand the significance of the decrease in the expression of Wnt10b and Wnt16. Nonetheless, these results seem to suggest that hnRNPI suppresses Wnt signaling in IECs through a mechanism independent of downregulating stromal Wnt ligands.

As an RNA-binding protein, hnRNPI exerts its function by controlling post-transcriptional events. Its effects on signaling pathways are highly context- and species- specific. hnRNPI inhibits Notch signaling in Drosophila wing disc [46] and during zebrafish intestinal homeostasis [20]. In mouse IECs, rather than inhibiting Notch signaling (S7 Fig), hnRNPI suppresses NF-κB and Wnt signaling. In the future, it will be of great interest to identify direct targets of hnRNPI and investigate the detailed molecular mechanisms by which hnRNPI influences major signaling pathways.

In summary, we report for the first time that hnRNPI-mediated post-transcriptional regulation is fundamentally important for establishing neonatal immune tolerance. The IEC-specific hnRNPI knockout mice represent a valuable animal model for studying regulatory mechanisms governing the establishment of neonatal immune tolerance at the post-transcriptional level.

## Materials and methods

### Animals

Generation of the *hnRNPI*^*flox/flox*^; *Villin*^*Cre/+*^ mice: hnRNPI targeted ES cells (KOMP Repository) were used for blastocyst injection (performed by the Transgenic Core Facility at the Research Institute at the Nationwide Children’s Hospital). Male chimeras were bred with wild type C57BL6 females for germline transmission. To obtain *hnRNPI*^*flox*^ mice, germline transmitted mice were bred with the ACT-FLPe (the Jackson Laboratory) mice to delete the *neo*-cassette. The *hnRNPI*^*flox*^ mice were crossed with the *villin-cre* mice (gift from Dr. Noah Shoyer) to generate the *hnRNPI*^*flox/flox*^; *Villin*^*Cre/+*^ mice. Primers used for genotyping the *hnRNPI* floxed allele are: F1: 5’–CCCATAACTGTCCATAGACC -3’, and B1: 5’ -TGTTGGTAATGCCAGCACAG -3’.

All mice with one exception used in this report are from the cross of the *hnRNPI*^*flox/flox*^; *Villin*^*Cre/+*^ mice with the *hnRNPI*^*flox/flox*^ mice. The *hnRNPI*^*flox/flox*^; *Villin*^*Cre/+*^ mice were used in the knockout group and the *hnRNPI*^*flox/flox*^ mice were used in the control group. An exception to this is the mice used in the adult weight statistical analysis. These mice were derived from the cross of the *hnRNPI*^*flox/+*^; *Villin*^*Cre/+*^ mice with the *hnRNPI*^*flox/flox*^ mice. In this experiment, the wild-type group includes the *hnRNPI*^*flox/+*^ mice and the *hnRNPI*^*flox/flox*^ mice, heterozygotes are the *hnRNPI*^*flox/+*^; *Villin*^*Cre/+*^ mice, and the knockout mice are the *hnRNPI*^*flox/flox*^; *Villin*^*Cre/+*^ mice.

### Histology, immunostaining, Alcian Blue-Periodic Acid Schiff (AB-PAS), histochemical staining, and fluorescence *in situ* hybridization

Colons were isolated, fixed, paraffin-embedded, and sectioned according to standard protocols. Intestine sections (5 μm) were processed for hematoxylin and eosin staining or for immunostaining. Immunohistochemistry was performed with R.T.U. vectastain kit (Vector Laboratories) with DAB substrate. Sections were counterstained lightly with Hematoxylin afterwards. For immunofluorescence staining, secondary antibodies used are goat anti-rabbit AlexaFluor 488 and donkey anti-rat AlexaFluor 594 (Invitrogen). Sections were counterstained with 4’,6-diamidino-2-phenylindole (DAPI). Primary antibodies used are: mouse anti-ki67 (BD Pharmingen, 550609), rat anti-CD4 (Ebioscience Inc, 14-9766-80), rat anti-Ly6G (BD Pharmingen, 551459), rabbit anti-F4/80 (Novus Biologicals Inc, NBP2-12506), rat anti-ZO-1 (Developmental Studies Hybridoma Bank, R26.4C), rabbit anti-hnRNPI (gift from Dr. Douglas Black), rabbit anti-p65 (*Santa Cruz* Biotechnology, sc-372), rabbit anti-p65 (Cell signaling, 8242), rabbit anti- β-catenin (gift from Dr. Peter Klein).

Goblet cell secreted mucins were identified by sequentially incubating deparaffinized sections in pH 2.5 alcian blue (1 hour), periodic acid (7 minutes) and Schiff's reagent (10 minutes). After the staining, acidic mucins are stained “blue” and neutral mucins are stained red.

Fluorescence *in situ* Hybridization (FISH) was performed to detect eubacteria infiltration in the colon. Paraffin sections (10 μm) were dewaxed and incubated with the commercially synthesized universal eubacterial probe EUB 338 (5′-GCTGCCTCCCGTAGGAGT-3′) conjugated with Alexa Fluor 488 as described [47]. A complimentary probe (5′-ACTCCTACGGGAGGCAGC-3′)) conjugated with Alexa Fluor 488 was used as a negative control. Sections were counterstained with DAPI afterwards.

Images were taken from a Compound microscope (Leica) with digital camera or a Nikon A1R confocal microscope and processed using Adobe Photoshop.

### Quantitative real-time PCR

Colonic epithelial cells were isolated as described [48]. RNAs were extracted from colonic epithelial cells isolated from adult mice and the postnatal day 4 (P4) neonatal mice or whole colon tissues from the P0, P1, P2, and P3 mice. RNA extraction was done using TRIzol reagent according to standard protocols. Real-time PCR reactions were performed blindly in triplicate or duplicate using SYBR green master mix (Applied Biosystem) on an Applied Biosystem's 7500 Real-time PCR system. PCR primers are: *Il6*: 5′- CCGGAGAGGAGACTTCACAG -3′ and 5′- CAGAATTGCCATTGCACAAC -3′; *Il1β*: 5′-CAACCAACAAGTGATATTCTCCATG-3′ and 5′-GATCCACACTCTCCAGCTGCA-3′*; Cxcl2/MIP-2*: 5′- GTGAACTGCGCTGTCAATGC -3′ and 5′- GCTTCAGGGTCAAGGCAAAC -3′; *Tnfα*: 5′-AGGGATGAGAAGTTCCCAAATG-3′ and 5′-TGTGAGGGTCTGGGCCATA-3′; *Ccl2*: 5′-AGGTCCCTGTCATGCTTCTG-3′ and 5′-TCTGGACCCATTCCTTCTTG-3’; *Cxcl1*: 5′-GCCAATGAGCTGCGCTGTCAATGC-3′ and 5′-CTTGGGGACACCCTTTTAGCATCTT-3’*; Tlr2*: 5′-GCTACCTGTGTGACTCTCCG-3′ and 5′- CGCCCACATCATTCTCAGGT-3′; *Tlr4*: 5′-GCTTTCACCTCTGCCTTCAC-3′ and 5′-AGGCGATACAATTCCACCTG-3′; *Tlr5*: 5′-CCAGCCCCGTGTTGGTAATA-3′ and 5′-TTTCTGAAAGCCCCTGGACC-3′; *IRAK1*: 5′-GGCTCAACTAGCTTGCTGCT-3′ and 5′-TAGTGCCTCCCTGGGTACAG-3′; and *Gapdh*: 5′-TTCTTGTGCAGTGCCAGCC-3′ and 5′-CACCGACCTTCACCATTTTGT-3′.

### Western blots

Isolated colonic epithelial cells from adult mice or whole colon tissues from the fetuses at embryonic day 19 or neonates at P0 or P3 were homogenized in lysis buffer. Protein lysates were cleared by spinning the samples twice at 4°C. Subsequently, samples were separated on SDS-PAGE and analyzed by western blotting as described [49]. Primary antibodies used are mouse anti-hnRNPI (Life Technologies, 324800), rabbit anti-IRAK1 (Santa Cruz, sc-7883), rabbit anti-Actin (Sigma, A2066). Membranes were incubated with HRP-linked secondary antibodies and developed using ECL prime (G&E Healthcare Life Sciences).

### Statistics

Differences between the knockout mice and the control groups were assessed for significance using a one-tailed unpaired Student *t*-test (Fig 3M). For data involving two variables, data were analyzed by two-way ANOVA using GraphPad Prism (Figs 6I, 6J, 7B and 7D) or R (Fig 2E). Log_2_ conversion was used in the figures where necessary (Figs 3M and 6I).

### Study approval

The use of mice in this research was approved by University of Illinois at Urbana-Champaign Animal Care and Use Committee (protocol #14240 and 14290).

## Supporting information

S1 Fig Colon histology of the IEC-specific hnRNPI knockout mice appears normal at P2. (A) and (B) H&E stained sections show the normal colonic epithelium in the knockout neonate at P2 (B). (C) to (F) Immunohistochemical staining with indicated antibodies shows similar numbers of immune cells in the lamina propria of the wild-type and knockout neonates at P2. (C) and (D) show CD4 positive T-cells, and (E) and (F) show macrophages. (G) to (H) Immunohistochemical staining with an anti-Ki67 antibody shows normal cell proliferation in the colonic epithelium of the knockout neonate at P2.
(TIF)

S2 Fig CD4 positive T-cells express hnRNPI. Double immunofluorescence staining using anti-hnRNPI and anti-CD4 antibodies shows hnRNPI protein localization in the CD4 positive T-cells in the wild-type and hnRNPI knockout colons (arrows). The number of hnRNPI-expressing CD4 positive T-cells in the lamina propria is increased in the knockout colon. Nuclei were counterstained with DAPI. The dotted lines indicate the borders of the crypts. The expression of hnRNPI is diminished in the crypt epithelial cells of the knockout mouse. WT, wild-type; KO, knockout. Scale bars, 50 μm.
(TIF)

S3 Fig Macrophages express hnRNPI. Double immunofluorescence staining using anti-hnRNPI and anti-F4/80 antibodies shows hnRNPI protein localization in macrophages in the wild-type and hnRNPI knockout colons. The number of hnRNPI-expressing macrophages in the lamina propria is increased in the knockout colon. Nuclei were counterstained with DAPI. The dotted line indicates the border of a crypt. hnRNPI expression is diminished in the crypt epithelial cells of the knockout mouse. WT, wild-type; KO, knockout. Scale bars, 50 μm.
(TIF)

S4 Fig Neutrophils express hnRNPI. Double immunofluorescence staining using anti-hnRNPI and anti-Ly6G antibodies shows hnRNPI expression in the neutrophils in the wild-type and hnRNPI knockout colon. Neutrophils were rarely detected in the wild-type colon and its number is increased in the knockout colon. Nuclei were counterstained with DAPI. The dotted lines indicate the borders of two crypts. hnRNPI expression is diminished in the crypt epithelial cells of the knockout mouse. WT, wild-type; KO, knockout. Scale bars, 50 μm.
(TIF)

S5 Fig α-SMA positive stromal cells express hnRNPI. Double immunofluorescence staining using anti-hnRNPI and anti-α-SMA antibodies shows hnRNPI expression in α-SMA positive stromal cells in the wild-type and hnRNPI knockout colon. The number of α-SMA and hnRNPI double positive stromal cells is not increased in the knockout colon. Nuclei were counterstained with DAPI. The dotted lines indicate the borders of three crypts. hnRNPI expression is diminished in the crypt epithelial cells of the knockout mouse. WT, wild-type; KO, knockout. Scale bars, 50 μm.
(TIF)

S6 Fig Expression of hnRNPI and Wnt ligands in the colon stroma. (A) to (C) Western blot results using protein extracts of the colonic epithelial and stromal fractions isolated from 3 wild-type and 3 knockout mice. Active β-catenin protein expression is increased in the colonic epithelium of the knockout mice (A). Increased hnRNPI protein expression in the colonic stroma of the same mice is shown in (B). The purity of the isolated colonic epithelial and stromal fractions is shown in (C). Vimentin and Cytokeratin serve as the control for isolation of colonic epithelial and stromal cells. (D) Real-time PCR results show the mRNA levels of *hnRNPI*, *wnt2b*, *wnt4*, *wnt5a*, *wnt5b*, *wnt10b*, *and wnt16* in the colonic stroma of the hnRNPI knockout mice and the control mice. A statistically significant increase in *hnRNPI* expression but not in *wnt2b*, *wnt4*, *wnt5a*, and *wnt5b* expression was detected in the colonic stroma of the knockout mice. *wnt10b* and *wnt16* display statistically significant decrease in their expression in the knockout colonic stroma. Each symbol in all graphs indicates gene expression level relative to *Gapdh* in the individual mouse. Bars show mean value. In the wild-type group, n = 6 mice; in the knockout group, n = 8 mice. * p < 0.05; ** p < 0.01. N.S., not significant.
(TIF)

S7 Fig Notch signaling activity is not elevated in the colonic epithelium of the hnRNPI-deficient mice. Western blot results using protein extracts of the colonic epithelial cells isolated from 2 wild-type and 2 knockout mice. The protein levels of hnRNPI are dramatically reduced in the colonic epithelial cells of the knockout mice while the protein levels of cleaved Notch1 are not increased. Actin served as the loading control. WT, wild-type; KO, knockout.
(TIF)

S1 Text Supporting materials and methods. (DOCX)

## References

[1] Tomkovich S, Jobin C (2016) Microbiota and host immune responses: a love-hate relationship. Immunology 147: 1–10. 10.1111/imm.12538 26439191 PMC4693877

[2] Sommer F, Backhed F (2013) The gut microbiota—masters of host development and physiology. Nat Rev Microbiol 11: 227–238. 10.1038/nrmicro2974 23435359

[3] Paun A, Danska JS (2015) Immuno-ecology: how the microbiome regulates tolerance and autoimmunity. Curr Opin Immunol 37: 34–39. 10.1016/j.coi.2015.09.004 26460968

[4] Karczewski J, Poniedzialek B, Adamski Z, Rzymski P (2014) The effects of the microbiota on the host immune system. Autoimmunity 47: 494–504. 10.3109/08916934.2014.938322 25019177

[5] Shanahan F, O'Toole PW (2014) Host-microbe interactions and spatial variation of cancer in the gut. Nat Rev Cancer 14: 511–512. 10.1038/nrc3765 25202783

[6] Sears CL, Garrett WS (2014) Microbes, microbiota, and colon cancer. Cell Host Microbe 15: 317–328. 10.1016/j.chom.2014.02.007 24629338 PMC4003880

[7] Peterson LW, Artis D (2014) Intestinal epithelial cells: regulators of barrier function and immune homeostasis. Nat Rev Immunol 14: 141–153. 10.1038/nri3608 24566914

[8] Kim YS, Ho SB (2010) Intestinal goblet cells and mucins in health and disease: recent insights and progress. Curr Gastroenterol Rep 12: 319–330. 10.1007/s11894-010-0131-2 20703838 PMC2933006

[9] Suzuki T (2013) Regulation of intestinal epithelial permeability by tight junctions. Cell Mol Life Sci 70: 631–659. 10.1007/s00018-012-1070-x 22782113 PMC11113843

[10] Turner JR (2009) Intestinal mucosal barrier function in health and disease. Nat Rev Immunol 9: 799–809. 10.1038/nri2653 19855405

[11] Abreu MT (2010) Toll-like receptor signalling in the intestinal epithelium: how bacterial recognition shapes intestinal function. Nat Rev Immunol 10: 131–144. 10.1038/nri2707 20098461

[12] de Kivit S, Tobin MC, Forsyth CB, Keshavarzian A, Landay AL (2014) Regulation of Intestinal Immune Responses through TLR Activation: Implications for Pro- and Prebiotics. Front Immunol 5: 60 10.3389/fimmu.2014.00060 24600450 PMC3927311

[13] Yu S, Gao N (2015) Compartmentalizing intestinal epithelial cell toll-like receptors for immune surveillance. Cell Mol Life Sci 72: 3343–3353. 10.1007/s00018-015-1931-1 26001904 PMC4534336

[14] Kawai T, Akira S (2010) The role of pattern-recognition receptors in innate immunity: update on Toll-like receptors. Nat Immunol 11: 373–384. 10.1038/ni.1863 20404851

[15] Cario E (2010) Toll-like receptors in inflammatory bowel diseases: a decade later. Inflamm Bowel Dis 16: 1583–1597. 10.1002/ibd.21282 20803699 PMC2958454

[16] Ospelt C, Gay S (2010) TLRs and chronic inflammation. Int J Biochem Cell Biol 42: 495–505. 10.1016/j.biocel.2009.10.010 19840864

[17] Fukata M, Vamadevan AS, Abreu MT (2009) Toll-like receptors (TLRs) and Nod-like receptors (NLRs) in inflammatory disorders. Semin Immunol 21: 242–253. 10.1016/j.smim.2009.06.005 19748439

[18] Lotz M, Gutle D, Walther S, Menard S, Bogdan C, et al (2006) Postnatal acquisition of endotoxin tolerance in intestinal epithelial cells. J Exp Med 203: 973–984. 10.1084/jem.20050625 16606665 PMC2118301

[19] Chassin C, Kocur M, Pott J, Duerr CU, Gutle D, et al (2010) miR-146a mediates protective innate immune tolerance in the neonate intestine. Cell Host Microbe 8: 358–368. 10.1016/j.chom.2010.09.005 20951969

[20] Yang J, Chan CY, Jiang B, Yu X, Zhu GZ, et al (2009) hnRNP I inhibits Notch signaling and regulates intestinal epithelial homeostasis in the zebrafish. PLoS Genet 5: e1000363 10.1371/journal.pgen.1000363 19197356 PMC2629577

[21] Mei W, Lee KW, Marlow FL, Miller AL, Mullins MC (2009) hnRNP I is required to generate the Ca2+ signal that causes egg activation in zebrafish. Development 136: 3007–3017. 10.1242/dev.037879 19666827 PMC2723070

[22] Keppetipola N, Sharma S, Li Q, Black DL (2012) Neuronal regulation of pre-mRNA splicing by polypyrimidine tract binding proteins, PTBP1 and PTBP2. Crit Rev Biochem Mol Biol 47: 360–378. 10.3109/10409238.2012.691456 22655688 PMC3422667

[23] Romanelli MG, Diani E, Lievens PM (2013) New insights into functional roles of the polypyrimidine tract-binding protein. Int J Mol Sci 14: 22906–22932. 10.3390/ijms141122906 24264039 PMC3856098

[24] Takahashi H, Nishimura J, Kagawa Y, Kano Y, Takahashi Y, et al (2015) Significance of Polypyrimidine Tract-Binding Protein 1 Expression in Colorectal Cancer. Mol Cancer Ther 14: 1705–1716. 10.1158/1535-7163.MCT-14-0142 25904505

[25] Thorsen K, Sorensen KD, Brems-Eskildsen AS, Modin C, Gaustadnes M, et al (2008) Alternative splicing in colon, bladder, and prostate cancer identified by exon array analysis. Mol Cell Proteomics 7: 1214–1224. 10.1074/mcp.M700590-MCP200 18353764

[26] Llorian M, Schwartz S, Clark TA, Hollander D, Tan LY, et al (2010) Position-dependent alternative splicing activity revealed by global profiling of alternative splicing events regulated by PTB. Nat Struct Mol Biol 17: 1114–1123. 10.1038/nsmb.1881 20711188 PMC2933513

[27] Gardina PJ, Clark TA, Shimada B, Staples MK, Yang Q, et al (2006) Alternative splicing and differential gene expression in colon cancer detected by a whole genome exon array. BMC Genomics 7: 325 10.1186/1471-2164-7-325 17192196 PMC1769375

[28] Misquitta-Ali CM, Cheng E, O'Hanlon D, Liu N, McGlade CJ, et al (2011) Global profiling and molecular characterization of alternative splicing events misregulated in lung cancer. Mol Cell Biol 31: 138–150. 10.1128/MCB.00709-10 21041478 PMC3019846

[29] Xue Y, Zhou Y, Wu T, Zhu T, Ji X, et al (2009) Genome-wide analysis of PTB-RNA interactions reveals a strategy used by the general splicing repressor to modulate exon inclusion or skipping. Mol Cell 36: 996–1006. 10.1016/j.molcel.2009.12.003 20064465 PMC2807993

[30] Xue Y, Ouyang K, Huang J, Zhou Y, Ouyang H, et al (2013) Direct conversion of fibroblasts to neurons by reprogramming PTB-regulated microRNA circuits. Cell 152: 82–96. 10.1016/j.cell.2012.11.045 23313552 PMC3552026

[31] Wang C, Norton JT, Ghosh S, Kim J, Fushimi K, et al (2008) Polypyrimidine tract-binding protein (PTB) differentially affects malignancy in a cell line-dependent manner. J Biol Chem 283: 20277–20287. 10.1074/jbc.M803682200 18499661 PMC2459264

[32] Wollerton MC, Gooding C, Robinson F, Brown EC, Jackson RJ, et al (2001) Differential alternative splicing activity of isoforms of polypyrimidine tract binding protein (PTB). RNA 7: 819–832. 11421360 10.1017/s1355838201010214 PMC1370133

[33] Madison BB, Dunbar L, Qiao XT, Braunstein K, Braunstein E, et al (2002) Cis elements of the villin gene control expression in restricted domains of the vertical (crypt) and horizontal (duodenum, cecum) axes of the intestine. J Biol Chem 277: 33275–33283. 10.1074/jbc.M204935200 12065599

[34] Jensen LE, Whitehead AS (2001) IRAK1b, a novel alternative splice variant of interleukin-1 receptor-associated kinase (IRAK), mediates interleukin-1 signaling and has prolonged stability. J Biol Chem 276: 29037–29044. 10.1074/jbc.M103815200 11397809

[35] Su J, Richter K, Zhang C, Gu Q, Li L (2007) Differential regulation of interleukin-1 receptor associated kinase 1 (IRAK1) splice variants. Mol Immunol 44: 900–905. 10.1016/j.molimm.2006.03.021 16690127

[36] Yanagisawa K, Tago K, Hayakawa M, Ohki M, Iwahana H, et al (2003) A novel splice variant of mouse interleukin-1-receptor-associated kinase-1 (IRAK-1) activates nuclear factor-kappaB (NF-kappaB) and c-Jun N-terminal kinase (JNK). Biochem J 370: 159–166. 10.1042/BJ20021218 12418963 PMC1223149

[37] Rao N, Nguyen S, Ngo K, Fung-Leung WP (2005) A novel splice variant of interleukin-1 receptor (IL-1R)-associated kinase 1 plays a negative regulatory role in Toll/IL-1R-induced inflammatory signaling. Mol Cell Biol 25: 6521–6532. 10.1128/MCB.25.15.6521-6532.2005 16024789 PMC1190355

[38] Cho S, Kim JH, Back SH, Jang SK (2005) Polypyrimidine tract-binding protein enhances the internal ribosomal entry site-dependent translation of p27Kip1 mRNA and modulates transition from G1 to S phase. Mol Cell Biol 25: 1283–1297. 10.1128/MCB.25.4.1283-1297.2005 15684381 PMC548013

[39] Taniguchi K, Ito Y, Sugito N, Kumazaki M, Shinohara H, et al (2015) Organ-specific PTB1-associated microRNAs determine expression of pyruvate kinase isoforms. Sci Rep 5: 8647 10.1038/srep08647 25721733 PMC4342556

[40] Taniguchi K, Sakai M, Sugito N, Kumazaki M, Shinohara H, et al (2016) PTBP1-associated microRNA-1 and -133b suppress the Warburg effect in colorectal tumors. Oncotarget 7: 18940–18952. 10.18632/oncotarget.8005 26980745 PMC4951342

[41] Engels B, Jannot G, Remenyi J, Simard MJ, Hutvagner G (2012) Polypyrimidine tract binding protein (hnRNP I) is possibly a conserved modulator of miRNA-mediated gene regulation. PLoS One 7: e33144 10.1371/journal.pone.0033144 22427970 PMC3302860

[42] Lustig Y, Barhod E, Ashwal-Fluss R, Gordin R, Shomron N, et al (2014) RNA-binding protein PTB and microRNA-221 coregulate AdipoR1 translation and adiponectin signaling. Diabetes 63: 433–445. 10.2337/db13-1032 24130336

[43] Gregorieff A, Pinto D, Begthel H, Destree O, Kielman M, et al (2005) Expression pattern of Wnt signaling components in the adult intestine. Gastroenterology 129: 626–638. 10.1016/j.gastro.2005.06.007 16083717

[44] Farin HF, Van Es JH, Clevers H (2012) Redundant sources of Wnt regulate intestinal stem cells and promote formation of Paneth cells. Gastroenterology 143: 1518–1529 e1517 10.1053/j.gastro.2012.08.031 22922422

[45] Kabiri Z, Greicius G, Madan B, Biechele S, Zhong Z, et al (2014) Stroma provides an intestinal stem cell niche in the absence of epithelial Wnts. Development 141: 2206–2215. 10.1242/dev.104976 24821987

[46] Dansereau DA, Lunke MD, Finkielsztein A, Russell MA, Brook WJ (2002) Hephaestus encodes a polypyrimidine tract binding protein that regulates Notch signalling during wing development in Drosophila melanogaster. Development 129: 5553–5566. 12421697 10.1242/dev.00153

[47] Johansson ME, Phillipson M, Petersson J, Velcich A, Holm L, et al (2008) The inner of the two Muc2 mucin-dependent mucus layers in colon is devoid of bacteria. Proc Natl Acad Sci U S A 105: 15064–15069. 10.1073/pnas.0803124105 18806221 PMC2567493

[48] Egea L, McAllister CS, Lakhdari O, Minev I, Shenouda S, et al (2013) GM-CSF produced by nonhematopoietic cells is required for early epithelial cell proliferation and repair of injured colonic mucosa. J Immunol 190: 1702–1713. 10.4049/jimmunol.1202368 23325885 PMC3563922

[49] Yang J, Wu J, Tan C, Klein PS (2003) PP2A:B56epsilon is required for Wnt/beta-catenin signaling during embryonic development. Development 130: 5569–5578. 10.1242/dev.00762 14522869

