# Supplementary figures and images for: hnRNP I regulates neonatal immune adaptation and prevents colitis and colorectal cancer
Source: PLoS Genet. 2017 Mar 15;13(3):e1006672. doi: 10.1371/journal.pgen.1006672 (PMC5371387; doi:10.1371/journal.pgen.1006672)

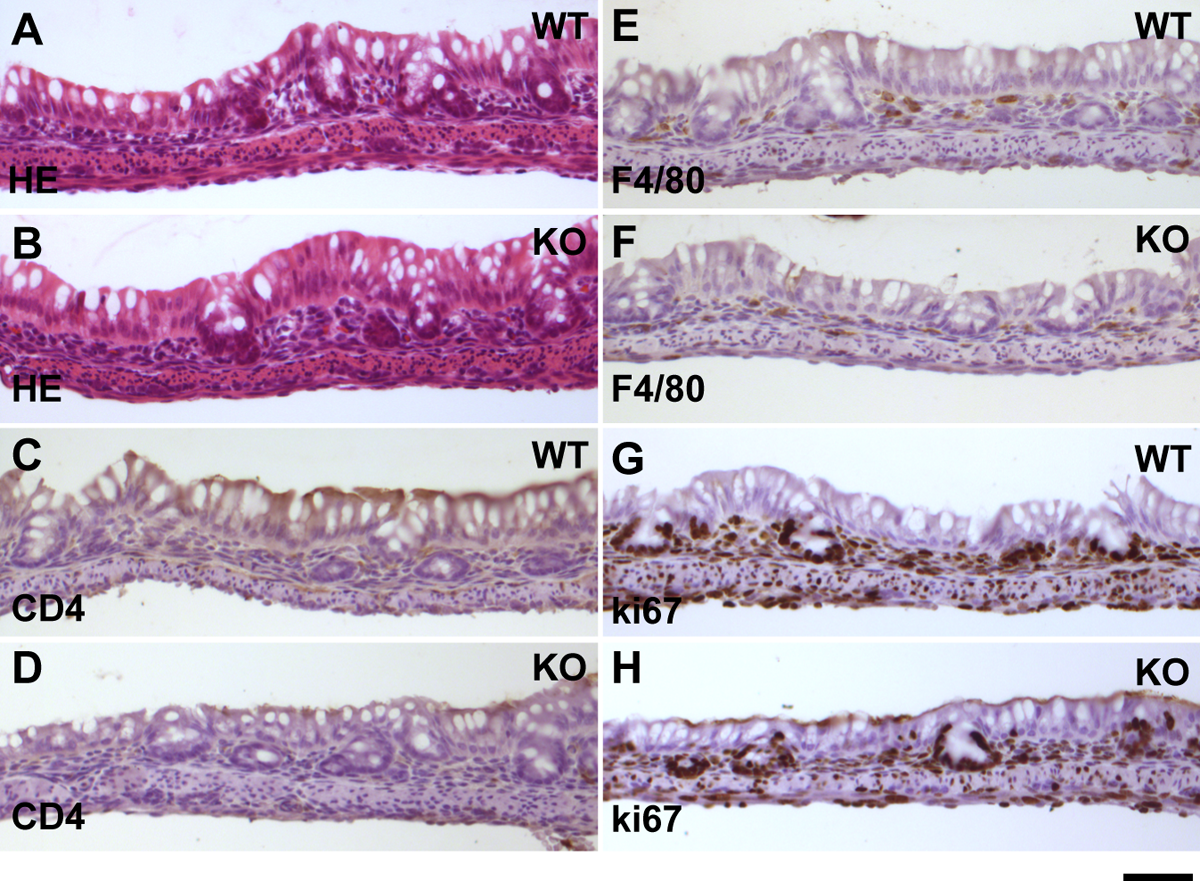

Supplement: S1 Fig — (A) and (B) H&E stained sections show the normal colonic epithelium in the knockout neonate at P2 (B). (C) to (F) Immunohistochemical staining with indicated antibodies shows similar numbers of immune cells in the lamina propria of the wild-type and knockout neonates at P2. (C) and (D) show CD4 positive T-cells, and (E) and (F) show macrophages. (G) to (H) Immunohistochemical staining with an anti-Ki67 antibody shows normal cell proliferation in the colonic epithelium of the knockout neonate at P2. (TIF) [file pgen.1006672.s001.tif]

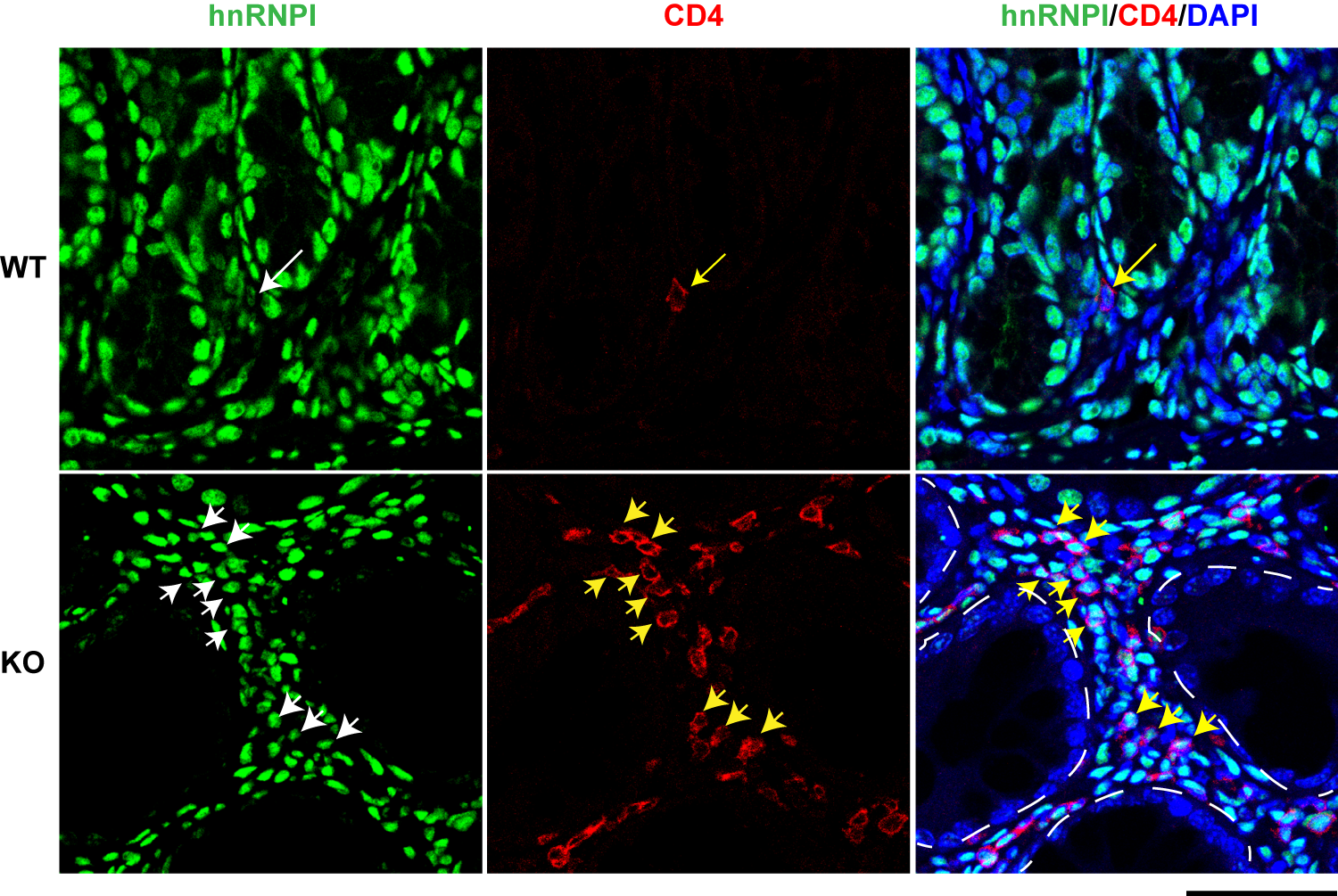

Supplement: S2 Fig — Double immunofluorescence staining using anti-hnRNPI and anti-CD4 antibodies shows hnRNPI protein localization in the CD4 positive T-cells in the wild-type and hnRNPI knockout colons (arrows). The number of hnRNPI-expressing CD4 positive T-cells in the lamina propria is increased in the knockout colon. Nuclei were counterstained with DAPI. The dotted lines indicate the borders of the crypts. The expression of hnRNPI is diminished in the crypt epithelial cells of the knockout mouse. WT, wild-type; KO, knockout. Scale bars, 50 μm. (TIF) [file pgen.1006672.s002.tif]

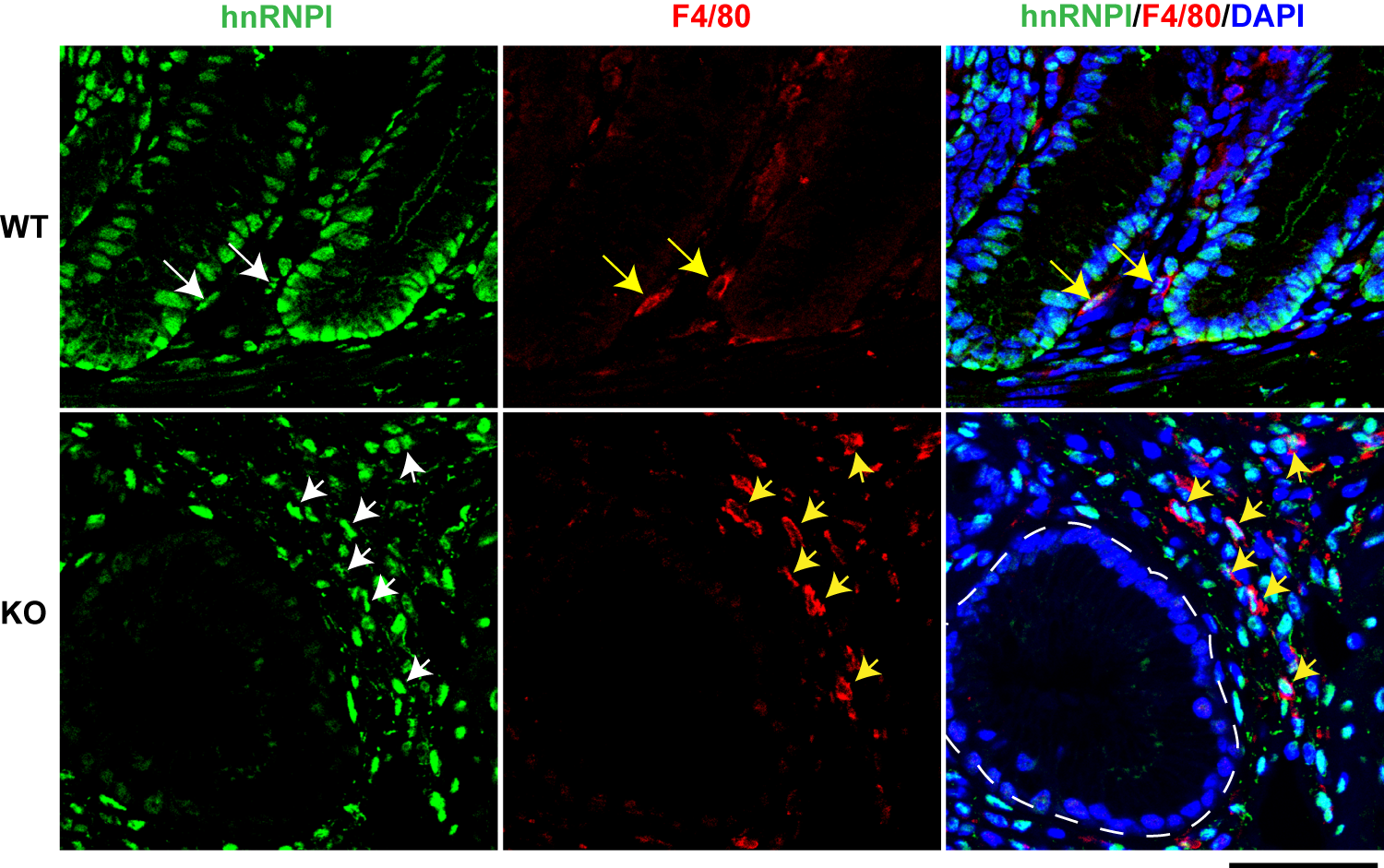

Supplement: S3 Fig — Double immunofluorescence staining using anti-hnRNPI and anti-F4/80 antibodies shows hnRNPI protein localization in macrophages in the wild-type and hnRNPI knockout colons. The number of hnRNPI-expressing macrophages in the lamina propria is increased in the knockout colon. Nuclei were counterstained with DAPI. The dotted line indicates the border of a crypt. hnRNPI expression is diminished in the crypt epithelial cells of the knockout mouse. WT, wild-type; KO, knockout. Scale bars, 50 μm. (TIF) [file pgen.1006672.s003.tif]

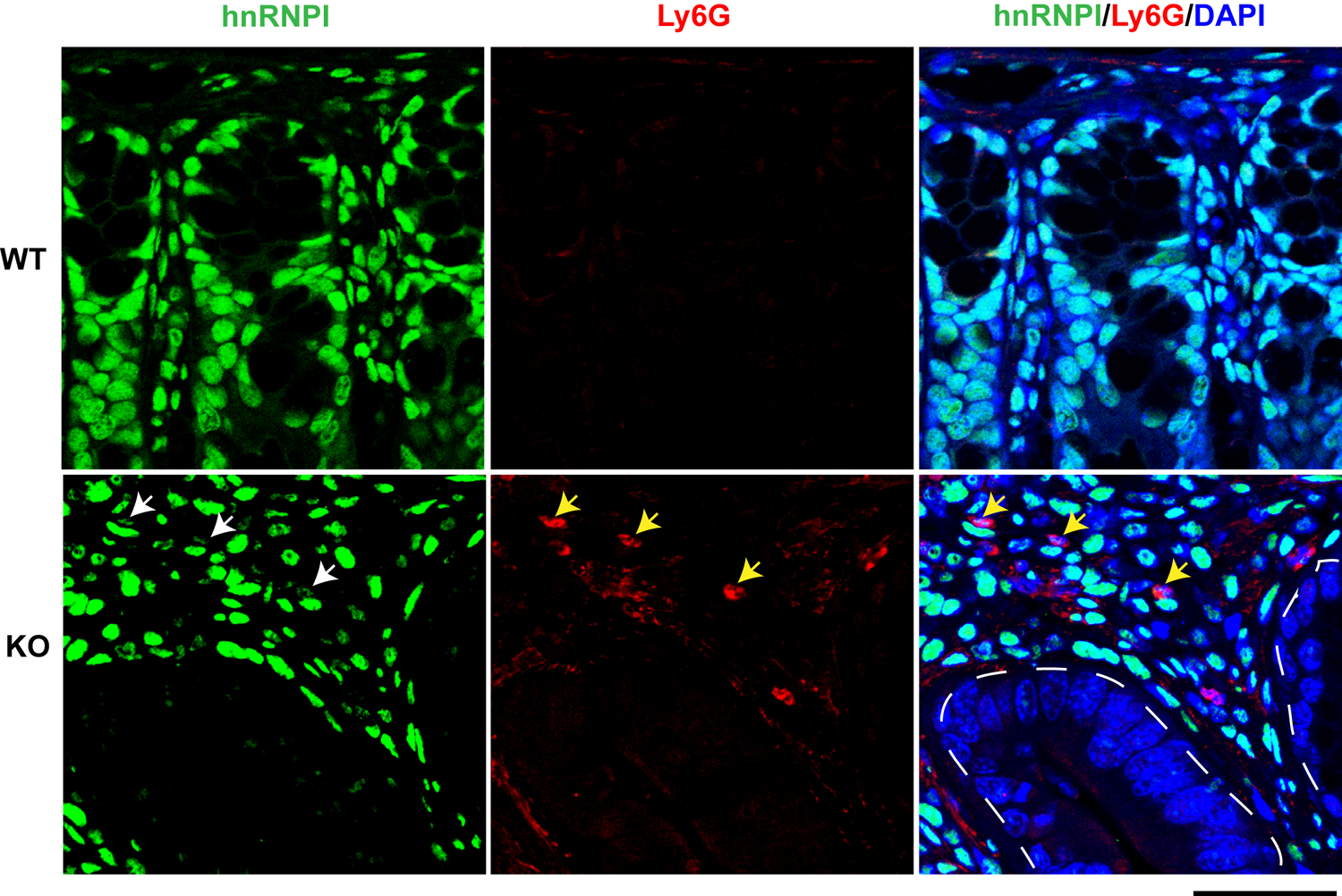

Supplement: S4 Fig — Double immunofluorescence staining using anti-hnRNPI and anti-Ly6G antibodies shows hnRNPI expression in the neutrophils in the wild-type and hnRNPI knockout colon. Neutrophils were rarely detected in the wild-type colon and its number is increased in the knockout colon. Nuclei were counterstained with DAPI. The dotted lines indicate the borders of two crypts. hnRNPI expression is diminished in the crypt epithelial cells of the knockout mouse. WT, wild-type; KO, knockout. Scale bars, 50 μm. (TIF) [file pgen.1006672.s004.tif]

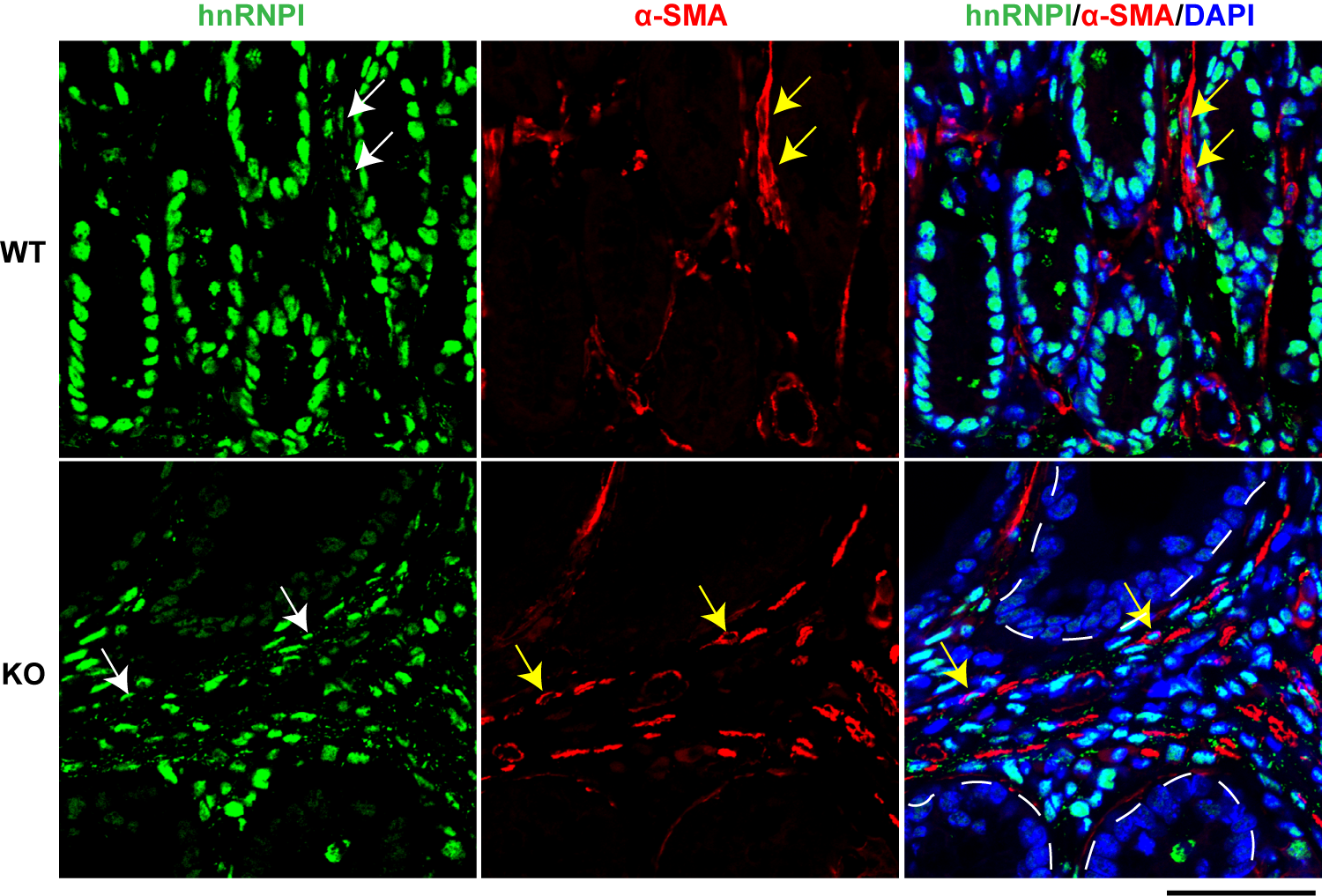

Supplement: S5 Fig — Double immunofluorescence staining using anti-hnRNPI and anti-α-SMA antibodies shows hnRNPI expression in α-SMA positive stromal cells in the wild-type and hnRNPI knockout colon. The number of α-SMA and hnRNPI double positive stromal cells is not increased in the knockout colon. Nuclei were counterstained with DAPI. The dotted lines indicate the borders of three crypts. hnRNPI expression is diminished in the crypt epithelial cells of the knockout mouse. WT, wild-type; KO, knockout. Scale bars, 50 μm. (TIF) [file pgen.1006672.s005.tif]

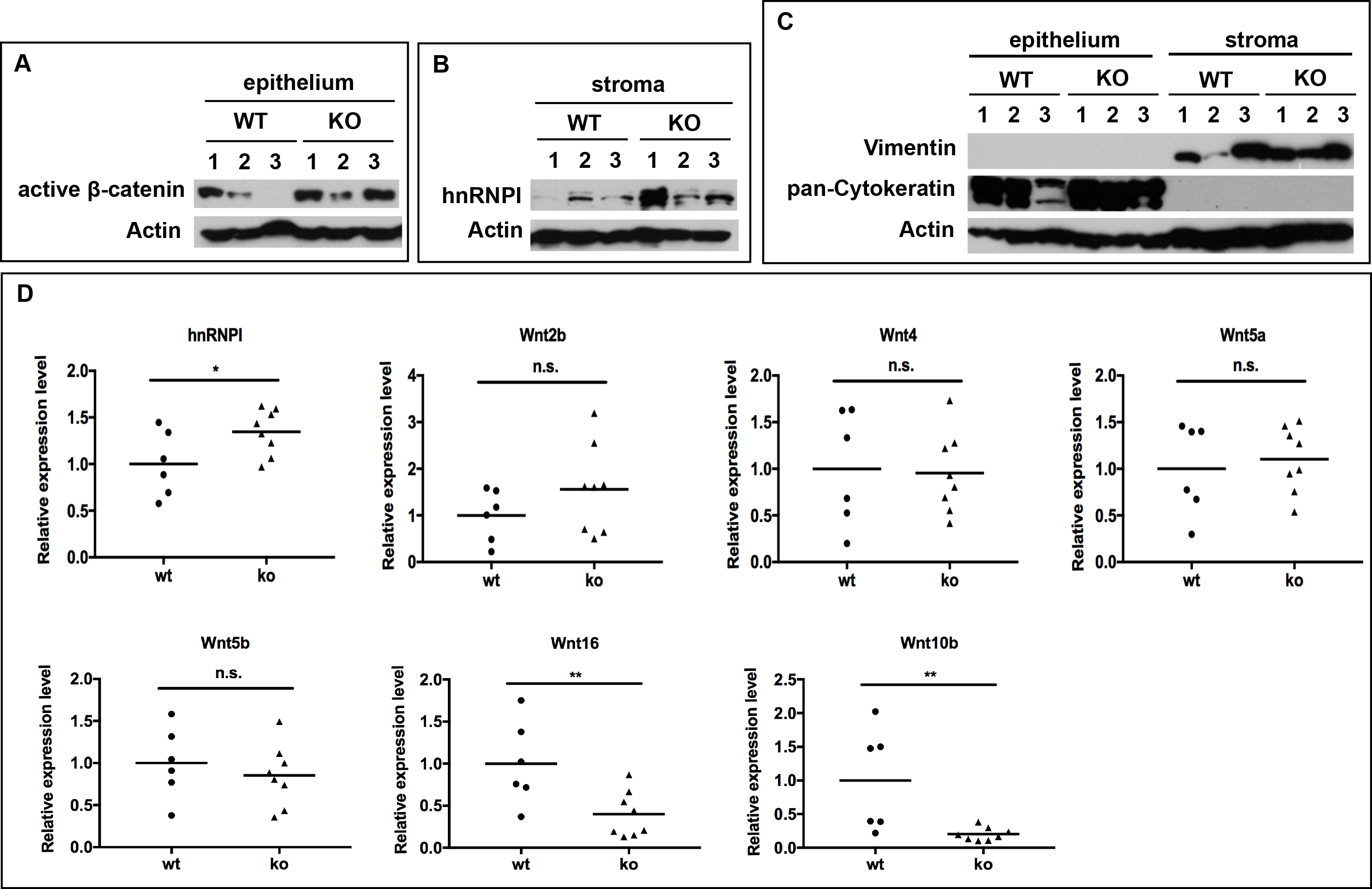

Supplement: S6 Fig — (A) to (C) Western blot results using protein extracts of the colonic epithelial and stromal fractions isolated from 3 wild-type and 3 knockout mice. Active β-catenin protein expression is increased in the colonic epithelium of the knockout mice (A). Increased hnRNPI protein expression in the colonic stroma of the same mice is shown in (B). The purity of the isolated colonic epithelial and stromal fractions is shown in (C). Vimentin and Cytokeratin serve as the control for isolation of colonic epithelial and stromal cells. (D) Real-time PCR results show the mRNA levels of hnRNPI, wnt2b, wnt4, wnt5a, wnt5b, wnt10b, and wnt16 in the colonic stroma of the hnRNPI knockout mice and the control mice. A statistically significant increase in hnRNPI expression but not in wnt2b, wnt4, wnt5a, and wnt5b expression was detected in the colonic stroma of the knockout mice. wnt10b and wnt16 display statistically significant decrease in their expression in the knockout colonic stroma. Each symbol in all graphs indicates gene expression level relative to Gapdh in the individual mouse. Bars show mean value. In the wild-type group, n = 6 mice; in the knockout group, n = 8 mice. * p < 0.05; ** p < 0.01. N.S., not significant. (TIF) [file pgen.1006672.s006.tif]

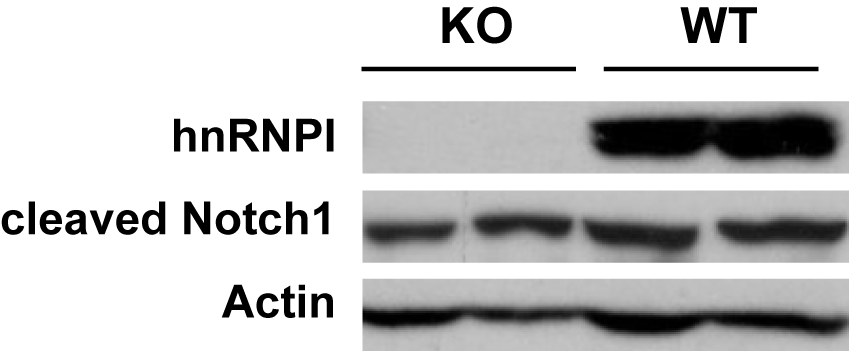

Supplement: S7 Fig — Western blot results using protein extracts of the colonic epithelial cells isolated from 2 wild-type and 2 knockout mice. The protein levels of hnRNPI are dramatically reduced in the colonic epithelial cells of the knockout mice while the protein levels of cleaved Notch1 are not increased. Actin served as the loading control. WT, wild-type; KO, knockout. (TIF) [file pgen.1006672.s007.tif]
