# Supplementary material for: hnRNP I regulates neonatal immune adaptation and prevents colitis and colorectal cancer
Source: PLoS Genet. 2017 Mar 15;13(3):e1006672. doi: 10.1371/journal.pgen.1006672 (PMC5371387; doi:10.1371/journal.pgen.1006672)
Supplement: S1 Text — (DOCX) [file pgen.1006672.s008.docx]

**Supporting Materials and Methods**

**Histology and Immunostaining**

Immunohistochemistry was performed as described in Materials and Methods. For immunofluorescence, primary antibodies used are: mouse anti-ki67 (BD Pharmingen, 550609), rat anti-CD4 (Ebioscience Inc, 14-9766-80), rat anti-Ly6G (BD Pharmingen, 551459), rabbit anti-F4/80 (Novus Biologicals Inc, NBP2-12506), rabbit anti-hnRNPI (gift from Dr. Douglas Black), goat anti-hnRNPI (Santa Cruz, sc-16547), rabbit anti-alpha smooth muscle Actin (Abcam, ab5694). Secondary antibodies used are goat anti-rabbit AlexaFluor 488, goat anti-rat AlexaFluor 568, donkey anti-rabbit AlexaFluor 488 and donkey anti-goat AlexaFluor 594. Sections were counterstained with 4’,6-diamidino-2-phenylindole (DAPI).

**Western Blots**

Intestinal epithelial and mesenchymal tissues were isolated as described [[1](#_ENREF_1)], and then were homogenized in the lysis buffer. Protein lysates were cleared by spinning the samples twice at 4°C. Subsequently, samples were separated on SDS-PAGE and analyzed by western blotting as described [[2](#_ENREF_2)]. Primary antibodies used are mouse anti-hnRNPI (Life Technologies, 324800), mouse anti-active-β-Catenin (Emd Millipore 05-665), rabbit anti-vimentin (Santa Cruz, sc-7557), mouse anti-Pan Cytokeratin (Sigma, C2931), rabbit anti-cleaved Notch1 (Cells Signaling, #4147), rabbit anti-actin (Sigma, A2066). Membranes were incubated with HRP-linked secondary antibodies and developed using ECL prime (G&E Healthcare Life Sciences).

### Quantitative Real-Time PCR

Intestinal mesenchymal tissues were isolated as described [[1](#_ENREF_1)]. RNA extraction was done using TRIzol reagent according to standard protocols. Real-time PCR reactions were performed blindly in triplicate or duplicate using SYBR green master mix (Applied Biosystem) on an Applied Biosystem's 7500 Real-time PCR system. PCR primers are hnRNPI: 5′- CAGAGGACGACCTCAAGAGC -3′ and 5′- TGGTGGACTTGGAAAAGGAC -3′; *wnt2b*: 5′-CACCCGGACTGATCTTGTCT -3′ and 5′- *TGTTTCTGCACTCCTTGCAC* -3′*;* *wnt4*: 5′- TGGACTCCCTCCCTGTCTTTGGGA -3′ and 5′- TCCTGACCACTGGAAGCCCTGTG -3′*; wnt5a*: 5′- CACGCTATACCAACTCCTCTGC -3′ and 5′- AATATTCCAATGGGCTTCTTCATGGC -3′; wnt5b: 5′-GCTGACTGACGCCAACTCCT -3′ and 5′-CTCCCTCCCCGATGTAGGAC-3′; wnt10b: 5′- GGAAGGGTAGTGGTGAGCAA -3′ and 5′- CACTTCCGCTTCAGGTTTTC -3′; wnt16: 5′- CTGTGACACCACCTTGCAGA -3′ and 5′- CAGGTTTTCACAGCACAGGA -3′.

**Statistics**

Differences between the knockout mice and the control groups were assessed for significance using a one-tailed unpaired Student *t*-test.

**Supporting information references**

1. Farin HF, Van Es JH, Clevers H (2012) Redundant sources of Wnt regulate intestinal stem cells and promote formation of Paneth cells. Gastroenterology 143: 1518-1529 e1517.

2. Yang J, Wu J, Tan C, Klein PS (2003) PP2A:B56epsilon is required for Wnt/beta-catenin signaling during embryonic development. Development 130: 5569-5578.
